# Factors necessary for independent walking in patients with thalamic hemorrhage

**DOI:** 10.1186/s12883-017-0991-2

**Published:** 2017-12-08

**Authors:** Shigenori Hiraoka, Shinichiro Maeshima, Hideto Okazaki, Hirokazu Hori, Shinichiro Tanaka, Sayaka Okamoto, Reisuke Funahashi, Kei Yagihashi, Ikuko Fuse, Naoki Asano, Shigeru Sonoda

**Affiliations:** 1 Department of Rehabilitation Medicine II, School of Medicine, Fujita Health University, Tsu, Japan; 2 Department of Rehabilitation Medicine, Fujita Health University, Nanakuri Memorial Hospital, 114-2 Oodoricho, Tsu, Mie 514-1295 Japan

**Keywords:** Hemorrhage, Thalamus, Outcome, Rehabilitation, Ambulation

## Abstract

**Background:**

Thalamic hemorrhages cause motor paralysis, sensory impairment, and cognitive dysfunctions, all of which may significantly affect walking independence. We examined the factors related to independent walking in patients with thalamic hemorrhage who were admitted to a rehabilitation hospital.

**Methods:**

We evaluated 128 patients with thalamic hemorrhage (75 men and 53 women; age range, 40–93 years) who were admitted to our rehabilitation hospital. The mean duration from symptom onset to rehabilitation hospital admission was 27.2 ± 10.3 days, and the mean rehabilitation hospital stay was 71.0 ± 31.4 days. Patients’ neurological and cognitive functions were examined with the National Institutes of Health Stroke Scale (NIHSS) and Mini-Mental State Examination (MMSE), respectively. The relationship between patients’ scores on these scales and their walking ability at discharge from the rehabilitation hospital was analyzed. Additionally, a decision-tree analysis was used to create a model for predicting independent walking upon referral to the rehabilitation hospital.

**Results:**

Among the patients, 65 could walk independently and 63 could not. The two patient groups were significantly different in terms of age, duration from symptom onset to rehabilitation hospital admission, hematoma type, hematoma volume, neurological symptoms, and cognitive function. The decision-tree analysis revealed that the patient’s age, NIHSS score, MMSE score, hematoma volume, and presence of ventricular bleeding were factors that could predict independent walking.

**Conclusions:**

In patients with thalamic hemorrhage, the neurological symptoms, cognitive function, and neuroimaging factors at onset are useful for predicting independent walking.

## Background

Cerebral hemorrhage occurs in 18.5% of stroke patients and thalamic hemorrhage accounts for 26% of all cerebral hemorrhages [1]. The thalamus is a vital structure that has extensive neural connections with other structures, allowing it to send signals throughout the brain including to the cerebral cortex. As such, the thalamus is involved in sensory and motor signal relays and in the regulation of consciousness. Given its interconnectedness with other regions, thalamic hemorrhages can cause cognitive dysfunctions such as aphasia, unilateral neglect, and memory impairments, as well as motor paralysis and sensory disturbances. These deficits can greatly affect a patient’s ability to perform activities of daily living (ADLs) [2]. The prognosis of patients with thalamic hemorrhage varies depending on the patient’s age, neurological severity, hematoma location and size, complications, and treatment type.

In rehabilitation wards, patients undergo intensive treatment in the early stages after stroke to help decrease ADL impairments and hasten recovery. Upon returning home, the reacquisition of walking ability is a major focus for patients with stroke with disabilities. However, few reports discuss the factors related to walking in patients with thalamic hemorrhage [3]. Understanding these factors is important for predicting patient outcome and for efficiently and effectively advancing their rehabilitation program.

The information available upon discharge of the patient from the acute-care hospital may be useful for predicting whether a patient will be able to walk independently upon discharge from the rehabilitation hospital. Here, we analyzed the factors related to independent walking in patients with thalamic hemorrhage who were admitted to a rehabilitation hospital.

## Methods

### Patients

From April 2013 to March 2016, 181 patients with thalamic hemorrhage visited the rehabilitation department of our hospital. After excluding patients with a history of previous stroke, neurodegenerative disease, and unconsciousness, as well as those who underwent surgical treatment or tracheotomy, we finally enrolled 128 patients (75 men and 53 women) in our study. The present study was conducted with the approval of the ethics committee at our university. Written informed consent was obtained from all patients or their legally acceptable representatives following a thorough explanation of the study.

### Evaluations

**Fig. 1:**
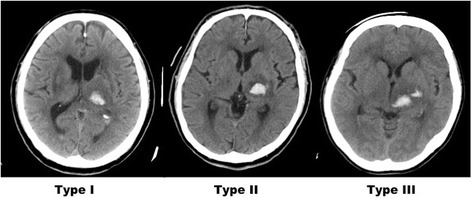
Computed tomography classification of thalamic hemorrhage. The hemorrhages were classified as follows: type I, hematoma localized in the thalamus; type II, hematoma extending into the internal capsule; and type III, hematoma extending into the midbrain

We evaluated the following items in our patients with thalamic hemorrhage: age, duration from symptom onset to rehabilitation hospital admission, classification for hematoma location on computed tomography (CT) images [4], side of the stroke focus, hematoma volume, ventricular bleeding (yes/no), and neurological and cognitive function. The hematoma type was classified as follows: type I, hematoma localized in the thalamus; type II, hematoma extending into the internal capsule; and type III, hematoma extending into the midbrain (Fig. 1). The hematoma volume was calculated using the CT images that were acquired upon admission to the acute-care hospital as follows: major axis of the hematoma × minor axis × height × 1/2 (mL) [5].

Neurological severity was evaluated using the National Institutes of Health Stroke Scale (NIHSS) [6], and cognitive function was evaluated using the Mini-Mental State Examination (MMSE) [7]. At rehabilitation hospital discharge, we assessed the patients’ functional ambulation category (FAC, Table 1) [8]. Here, patients were considered independent walkers if they had an FAC score ≥4 (i.e., they could walk independently on level ground but required assistance with stairs and slopes). We divided the patients into two groups based on the FAC at discharge, as follows: independent-walking group (FAC ≥4) and dependent-walking group (FAC <4).

**Table 1:** Functional ambulation classification

Clinician-completed tick box of five broad categories of walking ability.
The categories range from independent walking outside to non-functional walking.
Patients are rated based on the following categories:
0: Patient cannot walk or needs help from two or more persons.
1: Patient needs firm continuous support from one person who helps with carrying the weight and balance.
2: Patient needs the continuous or intermittent support of one person to help with balance and coordination.
3: Patient requires verbal supervision or stand-by help from one person without physical contact.
4: Patient can walk independently on level ground, but requires help on stairs, slopes, or uneven surfaces.
5: Patient can walk independently anywhere.

Holden MK et al. [8]

### Statistical analysis

Data were analyzed with JMP version 12.2.0 for Macintosh (SAS Institute Inc., Cary, NC). The Mann–Whitney U test was used to test for unpaired differences between the two groups, and the independence of two factors was examined with the chi-squared test. A decision-tree analysis (Partition) was used to show the relationships among the factors that lead to independent walking and their hierarchical classification. The classification model was developed based on methods first introduced by Breiman [9]. In our model, independent walking served as the response variable and age, sex, duration from symptom onset to rehabilitation hospital admission, lesion side, hematoma volume and type, ventricular bleeding, NIHSS score, MMSE score, aphasia, and unilateral neglect served as the explanatory variables. Statistical significance was set at p < 0.05.

## Results

### Demographic and clinical characteristics

The mean age of the 128 patients with thalamic hemorrhage was 67.6 ± 10.3 years (range, 40–93 years). The mean duration from symptom onset to hospitalization in the convalescent rehabilitation ward was 27.2 ± 10.3 days, and the mean duration of hospitalization was 71.0 ± 31.4 days. The mean hematoma volume was 8.1 ± 25.3 mL. Regarding the disease type, CT imaging revealed that six patients were type I, 87 patients were type II, and 18 patients were type III. Ventricular bleeding was observed in 86 patients.

### Comparisons between patients with and without independent walking

Among the 128 patients, 65 could walk independently (FAC ≥4; independent-walking group) at discharge and 63 could not (FAC <4; dependent-walking group). The two patient groups were significantly different in terms of age, duration from symptom onset to rehabilitation hospital admission, presence/absence of ventricular bleeding, hematoma volume, NIHSS scores, MMSE scores, and presence/absence of unilateral neglect (Table 2). Specifically, compared to the dependent-walking group, patients in the independent-walking group were younger and had shorter durations from symptom onset to rehabilitation hospital admission, less ventricular bleeding, smaller hematoma volumes, lower NIHSS scores, higher MMSE scores, and fewer instances of unilateral neglect.

**Table 2:** Comparisons between the independent- and dependent-walking groups

	Independent (FAC ≥4) n = 65	Dependent (FAC <4) n = 63	*p* value
Age (years)	65 (48–84)	71 (40–93)	0.0003
Sex, male/female	42/23	33/30	0.1595
Duration from symptom onset to rehabilitation hospital admission (days)	23 (11–48)	28 (11–57)	0.0045
Lesion side, right/left	30/35	33/30	0.4811
Hematoma volume (mL)	6 (0.9–17.3)	8.6 (0.3–25.3)	0.0038
Hematoma type, I/II/III	3/51/11	3/36/24	0.234
Ventricle bleeding, present/absent	38/27	48/15	0.0318
National Institutes of Health Stroke Scale	5 (1–17)	10 (2–23)	<0.0001
Mini-Mental State Examination (/30)	26 (8–30)	20 (6–30)	<0.0001
Aphasia, present/absent	20/45	22/41	0.617
Unilateral neglect, present/absent	9/56	24/39	0.0015

Data are presented as the number or as the mean and range

### Decision-tree analysis

**Fig. 2:**
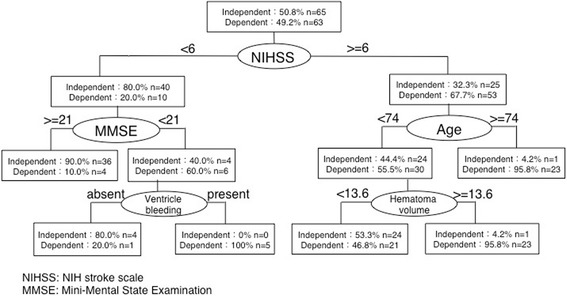
Decision-tree analysis for factors related to walking independence in patients with thalamic hemorrhage. The National Institutes of Health Stroke Scale (NIHSS) score was placed in the first tier and patients were divided based on their score. For patients with an NIHSS score <6, the second tier was divided based on the Mini-Mental State Examination (MMSE) score, while patients with an NIHSS score ≥6 were divided based on age. Regarding the third tier, patients with an MMSE score <21 were further divided according to the presence/absence of ventricular bleeding, while patients <74 years of age were divided according to the hematoma volume

Figure 2 depicts the decision tree. The positive classification rate in patients was 85.2%, and the error rate by cross validation was 0.042. The analysis revealed that the NIHSS score, MMSE score, and age were factors related to independent walking. In this model, the NIHSS score was placed in the first tier and patients were divided based on their score (<6 vs. ≥6). For patients with an NIHSS score <6, the second tier was divided based on the MMSE score (<21 vs. ≥21), while patients with an NIHSS score ≥6 were divided based on age (<74 vs. ≥74 years). Regarding the third tier, patients with an MMSE score <21 were further divided according to the presence/absence of ventricular bleeding, while patients <74 years of age were divided according to the hematoma volume.

## Discussion

The present study examined the relationships among evaluations performed upon admission to the rehabilitation hospital (i.e., after discharge from the acute-care hospital) and independent walking. Additionally, based on medical information frequently used in acute-care hospitals (CT images, NIHSS scores, MMSE scores), we performed a decision-tree analysis to investigate whether it is possible to predict independent walking upon admission to the rehabilitation hospital. Although a study indicated that patients’ functional outcomes could be predicted based on their NIHSS and MMSE scores at the time of admission to an acute-care hospital [10], few studies have investigated walking outcomes using the evaluation methods performed at acute-care hospital discharge [3].

We found that the dependent- and independent-walking groups were significantly different in terms of age, duration from symptom onset to rehabilitation hospital admission, presence/absence of ventricular bleeding, hematoma volume, NIHSS score, MMSE score, and presence/absence of unilateral neglect. Fukiishi et al. [3] noted that among the factors related to walking ability in patients with thalamic hemorrhage, the patient’s age, CT classification, hematoma volume, and state of consciousness at the time of admission to an acute-care hospital are particularly important. Indeed, evidence shows that the greater the hematoma volume, the poorer the functional outcome [11]. Unilateral neglect is also known to inhibit independent walking, and patients with unilateral neglect have lower ADL scores than do patients without neglect [2]. Consistent with these previous studies, the present study also revealed that age, hematoma volume, and neurological severity, as measured with the NIHSS, were related to independent walking in patients with thalamic hemorrhage. Reports from rehabilitation hospitals show that there is a strong relationship between an improved functional outcome at rehabilitation hospital discharge and the early initiation of rehabilitation services [12, 13]. Since no information on the rehabilitation interventions performed at the acute-care hospital was available in this study, this factor should be studied in the future.

Our decision-tree analysis revealed that the NIHSS and MMSE scores, hematoma volume, and presence/absence of ventricular bleeding were factors related to independent walking. By using the NIHSS and MMSE, which are standard, comprehensive evaluations performed at acute-care hospitals for patients with stroke, independent walking could be predicted in about 80% of the patients without using special evaluation scales like the Functional Independence Measure. Here, the MMSE was selected in the decision-tree analysis as a factor that was related to independent walking. This may be because patients with low MMSE scores need some assistance walking in the clinical setting, as they are unable to adapt to the environment and ultimately fall down. Such patients were given an FAC of 3 or less because they either required verbal supervision or stand-by help from one person. Therefore, patients with low MMSE scores often could not walk independently.

Our study suggests that independent walking can be estimated by adding the image-based diagnosis after stratification by NIHSS, MMSE, and age. Although the accuracy of predictions based only on neuroimaging data is not sufficiently high [14], the present study confirmed that neuroimaging data (hematoma volume and ventricular bleeding) are useful after stratification with neurologic symptoms using decision-tree analyses.

One limitation of the present study is that we utilized CT images. In order to improve the accuracy, future studies should consider the effects of microbleeds and asymptomatic cerebral infarctions using magnetic resonance images. Another limitation is that we did not consider the location of the lesion in the thalamus. This may be problematic, as different regions of the thalamus are involved in different functions. Specifically, the ventrolateral thalamus is involved in motor function, while the ventroposterolateral thalamus is involved in somatosensory function. Although we evaluated patients’ neurological deficits using the NIHSS, as well as the hematoma type, additional studies that consider the lesion location should be performed in the future to confirm our findings.

## Conclusion

Neuroimaging data, including the hematoma volume and presence of ventricular bleeding, along with age, neurological symptoms, and cognitive functions were useful for predicting the walking ability of patients with thalamic hemorrhage.

## Abbreviations

ADL: Activities of daily living
CT: Computed tomography
FAC: Functional ambulation category
MMSE: Mini-mental state examination
NIHSS: National Institutes of Health Stroke Scale
